# Elevated Risk of Overweight/Obesity-Related Markers and Low Muscular Fitness in Children Attending Public Schools in Chile

**DOI:** 10.3390/ijerph192114213

**Published:** 2022-10-31

**Authors:** Mónica Suárez-Reyes, Rodrigo Fernández-Verdejo, Gabriela Salazar

**Affiliations:** 1 Escuela de Ciencias de la Actividad Física, el Deporte y la Salud, Universidad de Santiago de Chile, Santiago 9170020, Chile; 2 Laboratorio de Fisiología del Ejercicio y Metabolismo (LABFEM), Escuela de Kinesiología, Facultad de Medicina, Universidad Finis Terrae, Santiago 7501015, Chile; 3 Instituto de Nutrición y Tecnología de los Alimentos (INTA), Universidad de Chile, Santiago 7830490, Chile

**Keywords:** socioeconomic level, childhood obesity, musculoskeletal health

## Abstract

In Chile, children of low socioeconomic status usually attend public schools and have few opportunities to engage in healthy behaviors. This may increase their risk of overweight/obesity and low muscular fitness. Therefore, we aimed to determine the association between the school type attended with overweight/obesity-related markers and the muscular fitness of children in Chile. We included 1410 children (6–13 years old) attending public, subsidized, or private schools. Overweight/obesity-related markers included BMI Z-scores, waist circumference, and body fat percentage. Muscular fitness assessment included handgrip strength and standing long jump. The odds ratios [95% CI] of overweight/obesity, elevated waist circumference, elevated body fat, low handgrip strength, and low standing long jump were compared between school types. Compared with boys attending public schools, those attending subsidized or private schools had lower odds ratios of low handgrip strength (0.63 [0.42–0.94] and 0.44 [0.25–0.78], respectively). Girls attending subsidized schools, compared with those in public schools, had lower odds of overweight/obesity (0.63 [0.44–0.90]) and of having low handgrip strength (0.51 [0.34–0.78]). Compared with girls in public schools, those attending private schools had lower odds (vs. public schools) of overweight/obesity (0.45 [0.28–0.74]), of having elevated body fat (0.53 [0.29–0.96]), and of having low standing long jump (0.41 [0.21–0.77]). The elevated risk of overweight/obesity-related markers and lower muscular fitness in children, particularly girls, attending public schools increase their current and future disease risk. This suggests that childhood socioeconomic status plays a central role in determining disease risk. Health-promoting interventions specifically focused on children from disadvantaged contexts are required.

## 1. Introduction

Healthy lifestyle habits help children maintain healthy body weight and physical/muscular fitness, which enhance physical and mental development throughout childhood and youth [1]. Adoption of these habits, however, may be affected by social factors such as socioeconomic status [2]. Children with low socioeconomic status have fewer opportunities to eat healthily and to engage in sufficient physical activity [3,4]. Consequently, in economically disadvantaged contexts, the prevalence of obesity is exacerbated, and children are less likely to have adequate physical/muscular fitness [5,6,7]. These data suggest that social context influences children’s current and long-term health by affecting lifestyle habits. Specifically, social context has been shown to influence their chances of developing cardiovascular risk factors such as high blood pressure, type 2 diabetes, and others [8,9,10]. 

Schools are where children spend most of their waking time. Schools offer different opportunities that impact children’s habits and, thus, their health [11]. In Chile, schools are split according to the type of funding as follows: (a) public, which are fully funded by the government; (b) subsidized, which have shared funding from the government and the families; and (c) private, which are fully funded by the families [12]. Public and subsidized schools are rated with an index of vulnerability according to the proportion of children in vulnerable situations. The index ranges from 0% to 100%, and serves as a criterion for receiving governmental support to feed students [13]. Private schools are not rated with the vulnerability index and do not receive governmental support to feed their students. This is because most students in private schools do not need such support. This suggests that children of low-to-medium socioeconomic status attend mostly public and subsidized schools, whereas children of high socioeconomic status attend mostly private schools. The type of school attended is thus considered an index of socioeconomic status in Chile. 

Because socioeconomic status influences overweight/obesity-related markers and the muscular fitness of children, this may also be the case for school types. Nevertheless, this has not been fully studied in Chile. We aimed to determine the association between the school type attended and overweight/obesity-related markers and the muscular fitness of children in Chile. Additionally, we explored the association between the school type attended and blood pressure. These results will allow us to identify the groups with the highest disease risk to help focus health-promotion strategies.

## 2. Materials and Methods

### 2.1. Design, Subjects, and Setting

We randomly selected schoolchildren from first to eighth grade of elementary education attending urban schools in the Metropolitan Region of Chile (n = 1410; 6–13 years old). The school types included were public, subsidized, or private. The proportion of children from each school type included in the study was similar to the national proportion of children attending each school type reported by the Chilean Education Ministry: 25% public, 59% subsidized, and 16% private [14]. The vulnerability index was >75% for public schools and 30–60% for subsidized schools. Private schools are not rated with the vulnerability index, as they have a low proportion of vulnerable students and therefore do not receive governmental support. This suggests that the school type was an adequate surrogate for socioeconomic status. Trained professionals conducted the measurements inside the schools. Before any procedure, we obtained the authorization of the school, parental consent, and child assent according to the statements of the Nutrition Institute and Food Technology of the University of Chile (Approval code Act No. 33, 2010).

### 2.2. Instruments and Data Collection

Overweight/obesity-related markers included measurements of the body mass index (BMI) Z-score, body fat, and waist circumference. Weight and height were measured with an electronic scale and a stadiometer (Seca^®^, Hamburg, Germany), respectively. BMI was calculated. The BMI Z-scores for 5- to 19-year-old children were used to categorize children as underweight (<–1.00), normal weight (–1.00 to 1.00), overweight (>1.00 to 2.00), or obesity (>2.00) according to categorization standards of the World Health Organization [15]. Skinfold thickness was measured with a Lange caliper at the triceps and subscapular sites. Body fat was estimated using the Slaughter equation [16]. There are no reference values for body fat in children. Therefore, we defined children >75th percentile for body fat (by age and sex) within our current sample as having elevated body fat. Table 1 shows the cutoffs used. Waist circumference was measured on the uppermost lateral border of the right ilium at the end of a normal expiration. To classify children as having elevated waist circumference and abdominal obesity, we used previously published reference values by age and sex calculated from African-American, European-American, and Mexican-American children [17].

Blood pressure was measured with a mercury sphygmomanometer. Children remained seated for 10 min before measurement. Systolic and diastolic blood pressures were then measured in the nondominant arm. We used previously published reference values by age, sex, and height to classify children as having elevated blood pressure and hypertension [18]. For this study, we assigned all children with elevated blood pressure or hypertension to the single category of elevated blood pressure.

Muscular fitness was measured through a handgrip strength test using an adjustable dynamometer (TKK 5101, Takei Scientific Instruments, Niigata, Japan). Muscular fitness was also assessed by the standing long jump test. Both tests have high reliability in children [19]. There are no reference values for these tests [6]. Therefore, we defined children ≤25th percentile (by age and sex) within our current sample as having low handgrip strength or low standing long jump. Table 1 shows the cutoffs used.

### 2.3. Statistics

Analyses were conducted, stratified by sex, using IBM SPSS Statistics version 26 (IBM Corp, Armonk, NY, USA), and considering *p* < 0.05 as statistically significant. Data for continuous variables are presented as medians (25th percentile–75th percentile). The Kolmogorov–Smirnov test was used to assess normal distribution of the data. Differences between school types (public, subsidized, and private) were tested with one-way ANOVA (for normally distributed data) or the Kruskal–Wallis test (for non-normally distributed data), followed by the Bonferroni post hoc test. Data for categorical variables are presented as percentages. Chi-square tests were used to assess the (unadjusted) association between school type and categorical variables.

Binary logistic regression models were used to compute the odds ratios and 95% confidence intervals (OR [95% CI]) for the (adjusted) associations between the school type attended and the overweight/obesity-related markers, blood pressure, and muscular fitness. The outcome variables were overweight/obesity, elevated waist circumference, elevated body fat, elevated blood pressure, low handgrip strength, and low standing long jump. The exposure variable was the school type (public, subsidized, or private), and public schools served as the reference category. Age and BMI Z-scores were considered potential confounders. The models were thus adjusted for either age, or age and BMI Z-scores, as indicated in the figure legend.

## 3. Results

Table 2 and Table 3 show the characteristics of boys and girls, respectively, by school type. Boys attending subsidized schools were taller than those attending public schools. Additionally, compared with subsidized and private schools, a larger proportion of boys attending public schools had low handgrip strength. No other differences were observed among boys. In girls, BMI Z-scores progressively decreased from public to subsidized to private schools. This led to a significant association between overweight/obesity prevalence and school type, with decreasing prevalence from public to subsidized to private schools. Girls attending private schools had the lowest waist circumference, which translated into the lowest prevalence of elevated waist circumference. These girls also had the lowest systolic blood pressure. The proportion of girls with low handgrip strength was associated with the school type. Girls attending subsidized schools had the lowest values. Finally, the standing long jump progressively increased from public to subsidized to private schools. This led to a significant association between the proportion of girls with low standing long jump and school type, with decreasing values from public to subsidized to private schools.

**Figure 1:**
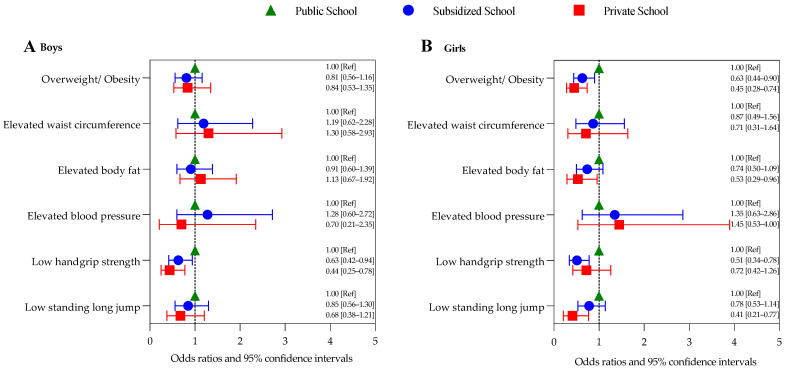
Odds ratios and 95% confidence intervals of having risky conditions according to school type in (**A**) boys, and (**B**) girls. Models for overweight/obesity and elevated body fat were adjusted for age; all other models were adjusted for age and body mass index Z-score. Ref, reference.

We then computed the OR [95% CI] of having risky conditions (i.e., overweight/obesity, elevated waist circumference, elevated body fat, elevated blood pressure, low handgrip strength, or low standing long jump) according to school type. Figure 1 shows the adjusted models. Compared with boys attending public schools, those attending subsidized or private schools had lower odds of having low handgrip strength (0.63 [0.42–0.94] and 0.44 [0.25–0.78], respectively). No other associations were observed in boys. Compared with girls attending public schools, those attending subsidized and private schools had lower odds of overweight/obesity (0.63 [0.44–0.90] and 0.45 [0.28–0.74], respectively). Girls attending private schools also had lower odds (vs. public) of having elevated body fat (0.53 [0.29–0.96]) and low standing long jump (0.41 [0.21–0.77]). Finally, girls attending subsidized schools had lower odds (vs. public) of having low handgrip strength (0.51 [0.34–0.78]).

## 4. Discussion

We observed some associations between the school type attended and certain overweight/obesity-related markers and indexes of muscular fitness in children from Chile. The associations were mostly observed in girls. In general, girls attending public schools showed elevated risk of overweight/obesity-related markers and lower muscular fitness compared with those attending subsidized or private schools. These results highlight the influence of social context and economic status on the current, and probably future, children’s health. 

### 4.1. Overweight/Obesity-Related Markers

Childhood obesity is a common phenomenon in Chile. The prevalence of overweight/obesity in Chilean children (≥5 years old) has been estimated at 30–44%, and it is higher in girls than boys [6,20,21]. Excess body weight accompanied by elevated body fat, especially in the waist, is associated with high disease risk in children and adolescents [17]. Therefore, BMI Z-scores, body fat, and waist circumference are used to identify and monitor children at risk. 

Compared with previous data, herein we observed a higher prevalence of overweight/obesity in all boys (>50%) as well as in girls attending public and subsidized schools (>46%). Only girls from our private school sample showed a similar prevalence (38.5%) to that previously reported in Chile. In boys, the school type was not associated with BMI Z-scores or prevalence of overweight/obesity; indeed, boys from all school types had average BMI Z-scores values above 1.00, which is the cutoff for overweight. This indicates that the boys in our study are at high risk but that this is independent of the school type (a surrogate for socioeconomic status). In contrast, progressively lower BMI Z-scores values were observed in girls from public to subsidized to private schools. Girls attending public schools had average BMI Z-scores values above 1.00 (i.e., overweight), although this was not the case for girls attending subsidized and private schools. Overall, school type was associated with the prevalence of overweight/obesity in both unadjusted and adjusted analyses. These results suggest that school type (a surrogate for socioeconomic status) does influence BMI Z-scores in girls. Girls attending public schools (i.e., those of low socioeconomic status) are at highest risk. 

A previous study reported a 40% prevalence of overweight/obesity in Chilean children, with no differences according to socioeconomic status (i.e., poverty) [22]. This agrees with our results in boys but not in girls. Note, however, that that such previous study was conducted in southern Chile, where a higher prevalence of overweight/obesity has been reported [23], whereas our study was conducted in the capital region. Differences in the characteristics of the schools or other context-related characteristics (e.g., urbanization or climate) may explain the discrepancy.

Regarding waist circumference, previous studies in Chilean schoolchildren have reported similar values to those observed in our current study [21,24,25,26,27]. The prevalence of elevated waist circumference has been previously estimated at 30–37% [6,26]. This agrees with our results in boys and girls attending subsidized schools and in boys attending private schools. However, the prevalence of elevated waist circumference reached >40% in boys and girls attending public schools. In the unadjusted analyses, school type was associated with the prevalence of elevated waist circumference in girls. Nevertheless, the association vanished when computing the odds ratio adjusted for BMI Z-scores. This last observation suggests that the association in girls was driven by BMI Z-scores. 

We observed body fat percentage values of 21–24% in our children. Previously, Urrejola et al. [28] reported lower values (12–16%) in their sample of Chilean children. In contrast, Muros et al. [25], also in a sample of Chilean children, reported slightly higher values in boys (24.0%) and similar values in girls (23.7%) compared with our current study (21.6% in boys and 23.9% in girls). The characteristics of the sample or differences in the assessment methods may explain the variability in the results of these studies. Of note, there are no reference values for body fat in children. We thus considered those children >75th body fat percentile according to age and sex as having elevated body fat. The cutoff values we considered in our sample coincide with those previously used in English children [29]. In boys, there was neither a difference in body fat percentage between school types nor an association between school type and elevated body fat (unadjusted or adjusted analyses). In girls, there were no differences in body fat percentage between school types; however, the unadjusted analyses showed a borderline association (*p* = 0.089) between school type and elevated body fat. This association reached significance in the adjusted analyses. Girls attending private schools thus had 47% lower odds of having elevated body fat, independent of BMI Z-scores, compared with girls attending public schools. Together, our results highlight the influence of school type (a surrogate for socioeconomic status) on overweight/obesity-related markers (BMI Z-scores and body fat) of girls.

Previous studies have reported poorer overweight/obesity-related markers in children of low compared with high socioeconomic status [2,30]. Herein, we observed such an association only in girls. In contrast, Jiménez-Pavón et al. [9] observed such an association only in boys. The discrepancy may be explained by the surrogate for socioeconomic status considered. We used the type of school, whereas Jiménez-Pavón et al. [9] used the educational level of the mothers. Which surrogate is the best is currently unknown, but in the Chilean context, school type seems a fairly good surrogate. 

### 4.2. Blood Pressure

Elevated blood pressure at an early age is associated with higher risk of hypertension in adulthood [31]. In children in our study, the prevalence of elevated blood pressure was between 3.3% and 6.9%, and there were no differences based on school type. This prevalence agrees with what was previously reported in another sample of Chilean children (6.3%) [32]. In contrast, another study in Chile reported a higher prevalence (12–15%) of elevated blood pressure [33]. Considering that the characteristics of the sample are similar and the reference to define elevated blood pressure is the same, the variations could be explained by methodological differences or other factors. In any case, our current findings suggest that school type (a surrogate for socioeconomic status) does not influence blood pressure. 

### 4.3. Muscular Fitness

Muscular fitness is associated with several cardiovascular risk factors [34,35]. Consequently, including muscular fitness in health monitoring systems at young ages has been suggested. In the current study, we measured muscular fitness with handgrip strength and the standing long jump test.

Previous studies in Chilean schoolchildren have reported similar values of handgrip strength to those observed in our sample (15.0–17.5 kg) [25,36]. Notably, those studies included only schoolchildren from public and subsidized schools. Our study is the first to report values in children attending private schools in Chile. Because there are no reference values to identify children at risk for low handgrip strength, we, separating children by sex and age, considered those ≤25th percentile as having low handgrip strength. This percentile appears appropriate, as previous data indicate higher cardiovascular risk in children below this cutoff [37]. Of note, the strength values (in kilograms) at this cutoff are similar to or slightly higher than those in previous studies [36,38,39]. This suggests that the children in our study performed slightly better on this test. We did not find differences in handgrip strength between school types. However, the unadjusted analyses showed an association between the prevalence of low handgrip strength and the school type in boys and girls. Furthermore, the adjusted analyses revealed that boys from private and subsidized schools as well as girls from subsidized schools had lower odds of having low handgrip strength than their peers attending public schools. These findings suggest that school type (a surrogate for socioeconomic status) influences muscle strength in schoolchildren. 

We also measured muscle power of the lower body with the standing long jump test. The values obtained in our study were lower than those previously reported in Chilean children (girls 131 cm and boys 133 cm) [25]. Again, we used the 25th percentile (by sex and age) as a cutoff to classify children as having low standing long jump. The values at our 25th percentile are lower than the values at the 25th percentile of previous studies [37,39]. Thus, the performance on this test by the children in our study was lower than previously reported. The school type did not influence the standing long jump in boys. Notably, however, performance by girls was progressively better from public to subsidized to private schools. School type was thus associated with prevalence of low standing long jump in unadjusted analyses. Moreover, the adjusted analyses revealed that girls attending private schools had 59% lower odds of having low standing long jump than girls attending public schools. These findings reinforce, specifically in girls, the effects of school type on muscular strength.

Together, our results suggest that the higher the socioeconomic status, the better the muscular fitness of children. Furthermore, this association seems stronger among girls. Similar associations have been observed in many previous studies [2,6,9,40,41]. Physical activity is one of the determinants of muscular fitness. Children in disadvantaged contexts may have fewer opportunities to engage in physical activity, which might partially explain the association between socioeconomic status and muscular fitness. Moreover, economic status can be a limiting factor for undertaking programmed sports, which are the type of physical activity that mostly impacts muscular fitness [42]. 

### 4.4. Strengths and Limitations

The main strength of our study is the large sample of children randomly chosen from different school types in order to match the national proportion of children attending each school type in Chile. This sampling method increases the representativeness of the findings. The main limitation of our study is the use of school type as a surrogate for socioeconomic status. Socioeconomic status in Chile is commonly determined by measuring family income. Unfortunately, we did not have access to this information. Note, however, that the vulnerability indexes of the included schools supported the association between school type and socioeconomic status. Another limitation of our study is the use of the 25th and 75th percentiles within our current sample as cutoffs for those variables without reference values. This reduces the comparability with other studies. Finally, we did not collect data on dietary status, nutrition education, or levels of physical activity, which are factors that may help explain the observed associations. Future studies should identify mediators of the association between the school type attended and overweight/obesity-related markers and muscular fitness.

## 5. Conclusions

We showed that girls attending public schools have higher overweight/obesity-related markers and lower muscular fitness than girls attending subsidized or private schools. In boys, a similar pattern is observed for muscular fitness. Of note, children with the lowest socioeconomic statuses attend public schools. Thus, our results highlight the influence of socioeconomic status on these markers of current and future health. Children attending public schools, and especially girls, are the most vulnerable group to the development of health-risky conditions. Strategies to promote healthy lifestyle habits should consider these differences between school types and sex.

## Figures and Tables

**Table 1:** Cutoff values to determine the risk of elevated body fat, low handgrip strength, and low standing long jump by sex and age.

	Age (Years)								
	6.0–6.9	7.0–7.9	8.0–8.9	9.0–9.9	10.0–10.9	11.0–11.9	12.0–12.9	13.0–13.9	14.0–14.9
n (girls/boys)	61/55	80/70	92/76	92/89	80/94	101/98	88/98	93/89	26/28
Body fat at 75th percentile (%)									
Girls	23.2	25.6	26.1	27.1	27.2	30.6	31.1	32.1	33.1
Boys	22.5	22.5	27.4	25.6	29.7	30.1	30.0	28.9	25.9
Handgrip strength at 25th percentile (kg)									
Girls	8.5	9.9	11.1	12.2	14.5	16.7	18.5	19.9	20.5
Boys	9.0	10.3	11.8	12.9	14.8	17.0	20.0	23.6	25.3
Standing long jump at 25th percentile (cm)									
Girls	81.0	86.0	93.2	103.0	103.0	105.5	109.2	113.5	105.0
Boys	84.0	92.7	99.2	105.5	112.7	118.0	125.0	136.0	144.7

**Table 2:** Characteristics of boys by school type.

	All	Public	Subsidized	Private
*n*	697	173	403	121
Age (years)	10.6 (8.6–12.4)	10.3 (8.5–12.3)	10.8 (8.8–12.5)	10.1 (7.9–12.1)
Weight (kg)	39.7 (31.1–50.9)	38.6 (30.0–51.3)	40.5 (31.6–51.2)	37.8 (30.7–49.4)
Height (cm)	142.5 (131.8–154.8)	140.0 (128.9–152.2)	143.5 (133.0–156.6) *	141.1 (132.0–153.9)
BMI Z-scores	1.07 (0.22–1.92)	1.29 (0.25–2.08)	1.03 (0.19–1.90)	1.13 (0.20–1.68)
Overweight/obesity (%)	52.8	56.6	51.1	52.9
Waist circumference (cm)	67.3 (61.0–75.0)	67.8 (60.4–76.5)	67.3 (61.5–75.3)	66.1 (60.6–72.9)
Elevated waist circumference (%)	35.4	40.5	34.0	33.1
Body fat (%)	21.6 (15.8–28.0)	22.1 (15.3–27.8)	21.1 (15.9–28.1)	21.1 (16.4–28.0)
Elevated body fat (%)	24.4	24.9	23.3	27.3
Systolic blood pressure (mmHg)	98.0 (90.0–104.0)	98.0 (90.0–105.0)	98.0 (90.0–103.0)	97.0 (90.0–106.0)
Diastolic blood pressure (mmHg)	55.0 (50.0–60.0)	57.0 (50.0–60.0)	54.0 (50.0–60.0)	53.0 (49.0–60.0)
Elevated blood pressure (%)	6.3	6.9	6.9	3.3
Handgrip strength (kg)	17.0 (13.0–22.3)	16.5 (12.1–21.5)	17.4 (13.4–23.0)	17.0 (12.5–22.0)
Low handgrip strength (%)	26.7	33.5 ^#^	25.8 ^#^	19.8 ^#^
Standing long jump (cm)	125.0 (110–142)	123.0 (104.5–138.0)	127.0 (111.0–143.0)	124.0 (105.5–146.0)
Low standing long jump (%)	25.4	30.1	24.8	20.7

Data are medians (25th percentile–75th percentile) or percentages. BMI Z-scores, Z-score of body mass index; * *p* < 0.05 vs. public; ^#^ *p* < 0.05 for an association with school type.

**Table 3:** Characteristics of girls by school type.

	All	Public	Subsidized	Private
*n*	713	186	423	104
Age (years)	10.4 (8.4–12.2)	10.2 (8.2–12.3)	10.6 (8.6–12.3)	9.8 (8.2–12.2)
Weight (kg)	38.8 (30.1–49.3)	38.2 (30.5–49.3)	39.5 (30.5–50.1)	37.6 (28.3–46.9)
Height (cm)	141.8 (131.0–153.0)	139.3 (128.1–151.7)	143.5 (131.7–153.6)	141.4 (130.9–153.2)
BMI Z-scores	0.95 (0.14–1.68)	1.24 (0.47–1.90)	0.91 (0.06–1.66) **	0.61 (–0.07–1.36) **^$$^
Overweight/obesity (%)	48.0	57.7 ^##^	46.1 ^##^	38.5 ^##^
Waist circumference (cm)	66.3 (60.2–73.6)	67.0 (61.9–75.3)	66.8 (60.0–74.0)	63.7 (58.3–68.9) **^$^
Elevated waist circumference (%)	34.6	43.0 ^##^	33.8 ^##^	23.1 ^##^
Body fat (%)	23.9 (19.6–27.9)	23.9 (19.2–28.3)	24.4 (19.6–29.2)	23.0 (19.7–26.4)
Elevated body fat (%)	24.5	29.6 ^₸^	23.9 ^₸^	18.3 ^₸^
Systolic blood pressure (mmHg)	95.0 (90.0–102.0)	96.0 (88.0–101.0)	97.0 (90.0–103.0)	91.0 (88.0–100.0) ^$^
Diastolic blood pressure (mmHg)	54.0 (50.0–60.0)	53.0 (49.0–60.0)	55.0 (50.0–60.0)	55.0 (50.0–60.0)
Elevated blood pressure (%)	6.3	5.4	6.6	6.7
Handgrip strength (kg)	16.0 (12.2–21.3)	15.5 (11.8–19.7)	16.6 (12.6–21.9) ^&^	15.0 (11.5–20.1)
Low handgrip strength (%)	25.7	30.6 ^#^	22.2 ^#^	30.8 ^#^
Standing long jump (cm)	111.0 (97.0–125.0)	107.0 (93.0–120.0)	111.0 (98.0–124.0) *	117.5 (103.2–131.7) **^$^
Low standing long jump (%)	25.5	32.3 ^##^	25.3 ^##^	14.4 ^##^

Data are medians (25th percentile–75th percentile) or percentages. BMI Z-scores, Z-score of body mass index; ^&^ *p* < 0.10, * *p* < 0.05, ** *p* < 0.01 vs. public; ^$^ *p* < 0.05, ^$$^ *p* < 0.01 vs. subsidized; ^₸^ *p* < 0.10, ^#^ *p* < 0.05, ^##^ *p* < 0.01 for an association with school type.

## Data Availability

Not applicable.
